# Theoretical performance of progressive addition lenses with poorly measured individual parameters

**DOI:** 10.1111/opo.13088

**Published:** 2023-01-09

**Authors:** Eduardo Pascual, José A. Gómez‐Pedrero, José Alonso

**Affiliations:** ^1^ Indizen Optical Technologies S.L. C/ Suero de Quiñones Madrid Spain; ^2^ Applied Optics Complutense Group, Facultad de Óptica y Optometría Universidad Complutense de Madrid Madrid Spain

**Keywords:** individual parameters, progressive addition lenses, visual acuity

## Abstract

**Purpose:**

The aim of this paper was to present a theoretical study of how poorly measured individual parameters affect the optical performance of progressive addition lenses (PALs). Modern progressive lenses can be prescribed based on parameters such as vertex distance, pantoscopic and wrap angles. These parameters can be measured from the lens wearer using specific devices; however, not all of them can be measured with the same precision, and the impact of measurement errors on the lens performance is still unknown.

**Methods:**

Data from 1900 patients were used to simulate the performance of four PAL designs with different degrees of complexity: perfect individual design, individual design with induced errors in the individual parameters, optimised design and conventional/basic design. For each patient and design, a quality metric was calculated to describe the optical performance of the lens.

**Results:**

The design having the best performance was the perfect individual design, followed by the individual design with induced errors, the optimised design and finally the conventional/basic design.

**Conclusions:**

Individual designs with measurement errors have better optical performance than lenses with less complexity, such as the optimised or conventional designs. This knowledge is useful for the eye care professional to make informed choices when dispensing these lenses.

**Key points**

- The quality of measured individual parameters may affect the optical performance of personalised progressive addition lenses.
- The performance of customised progressive addition lenses is better than that of basic or conventional lenses even when the individual parameters are poorly obtained.
- Knowledge of how poorly obtained patient parameters affects the performance of progressive addition lenses may help eye care professionals while dispensing these lenses.

## INTRODUCTION

Progressive addition lenses (PALs) can be manufactured with different degrees of complexity. Conventional PALs are mass‐produced using semi‐finished lens blanks for each base curve and add power, resulting in a limited number of geometric designs. The spherical or astigmatic prescription is surfaced onto the back side of the lens using traditional surfacing with a spherical or toroidal surface. Although conventional PALs have been used for many years, they can suffer significant degradation in optical performance if the combination of the spherical or toroidal back surface and the semi‐finished progressive front surface is not optimal.^1^, ^2^, ^3^

For the past 20 years, the development of free‐form manufacturing has allowed the production of complex designs on a per lens basis, which may be used to create lenses with several levels of personalisation based on the patient's data. The most basic format, which we will call ‘basic design’, calculates the lens by superimposing a fixed progressive surface onto the spherocylindrical prescription of the user. This concept is very similar to a conventional design: oblique aberrations are not corrected in either design, and, therefore, both types may be expected to have similar performance.^4^ Free‐form manufacturing can produce lenses optimised for the anthropometric and fitting parameters of the wearers. Several individual (patient) parameters are considered, including naso‐pupillary distance, vertex distance, pantoscopic and wrap angles.^5^ Several studies have shown that this customised type of lens design performs better than conventional PALs.^2^, ^4^, ^6^, ^7^, ^8^

Among these patient measurements, the naso‐pupillary distance (also known as the monocular inter‐pupillary distance or monocular PD) is well known by eye care practitioners (ECPs), and a poorly measured naso‐pupillary distance or a poorly fitted lens can result in unwanted prism and non‐adaptation.^9^, ^10^ There are several devices available to ECPs to measure this parameter: from simple rulers and pupilometers to more sophisticated self‐centring machines.^11^, ^12^ Several studies have shown that most devices exhibit good precision and repeatability when measuring the naso‐pupillary distance,^12^, ^13^, ^14^ but other fitting parameters are not always measured as accurately. We will call these ‘individual parameters’, that is, vertex distance, pantoscopic and wrap angle. A lens design that considers these parameters is called an ‘individual design’. However, these measurements are not always quantified by ECPs. That is because conventional PALs did not require these parameters, and only in more recent years have lens manufacturers provided devices to measure them. Therefore, some lens manufacturers offer a design with an intermediate degree of complexity in which the position of wear is considered but using fixed values for the individual parameters. We will call this lens an ‘optimised design’, following the nomenclature used in previous work.^2^, ^8^

There are several devices available to measure these individual parameters, including rulers, mobile and tablet applications and self‐centring devices. Both Wesemann^11^ and Garcia‐Espinilla et al.^12^ showed good repeatability when measuring the naso‐pupillary distance, but worse repeatability for the individual parameters. This could affect both vision and lens adaptation for the user. Indeed, Wesemann suggested that the increased variability could be related to the subject's head posture when the measurements were taken.

The fact that individual parameters are more prone to measurement errors led us to question the actual optical performance of the individual lens design. Assuming that we know the ‘true’ individual parameter values, then individual designs calculated with these parameters would be optimal. We will call this the ‘perfect individual’ design. However, since these parameters are measured with some degree of error, then we will have an individual design calculated with incorrect values. The actual ‘individual’ design will not be optimal, and its performance will be affected by the measurement errors.

The question that arises is how much do these measurement errors affect the lens design. In other words, how large is the optical degradation from the ‘perfect individual design’ to the ‘individual design’, and then to the ‘optimised’ and ‘conventional/basic’ designs. In fact, the individual design could perform worse than the optimised design because the former is subject to measuring errors, whereas the latter is not. This uncertainty may lead one to disregard the individual design and only use the optimised one. Therefore, a quantitative knowledge of the effect of these errors on the optical performance of a customised PAL would be useful for the ECP to be able to make informed choices when dispensing these lenses. The aim of this study was to theoretically quantify the optical degradation of four progressive designs, that is, conventional, optimised, individual and the perfect individual design. First, we created a randomised set of prescriptions and individual parameters based on patient data. Second, we analysed and compared the four designs based on their performance.

## METHOD

**FIGURE 1:**
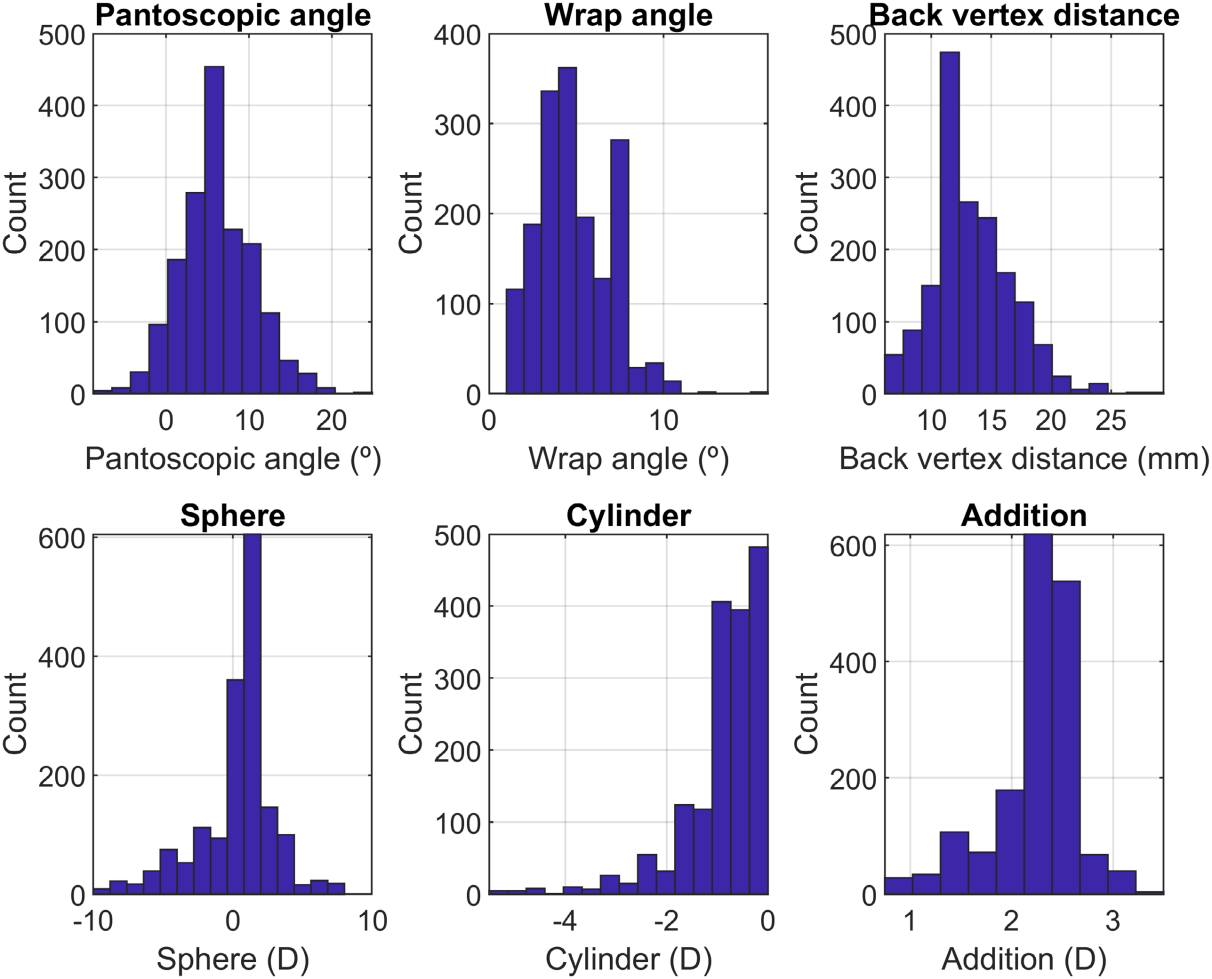
Data set showing pantoscopic angle, wrap angle, back vertex distance and patient's sphere, cylinder and near addition.

We considered a data set from 1900 patients comprising the spectacle prescription, naso‐pupillary distance, individual parameters and frame information. Although errors might be expected within these individual parameters, we assumed that the measurements reflect the ‘true’ values for each patient. Figure 1 shows the distribution of pantoscopic angle, wrap angle, vertex distance and sphere, cylinder and near addition power.

For each patient, we calculated four free‐form designs, namely:

1. Conventional/basic: A PAL in which the back surface blends the prescription of the patient with a progressive design.
2. Optimised: A PAL considering ray‐tracing of the lens‐eye system with minimisation of oblique aberrations assuming a fixed position of wear, with values of 8°, 5° and 12 mm for pantoscopic angle, wrap angle and vertex distance, respectively.
3. Perfect individual: A PAL calculated considering ray‐tracing of the lens‐eye system, with minimisation of oblique aberrations for the individual parameters. This is the ‘state‐of‐the‐art’ lens that best compensates for the eye, and represents the reference against which the optical quality of the other three designs is compared.
4. Individual: A PAL calculated considering ray‐tracing of the lens‐eye system, with minimisation of oblique aberrations considering that the user's individual parameters will include some errors. These errors are uniformly distributed: ±2° for tilt and ±1 mm for the vertex distance.

As an example, assume for a given user that the pantoscopic angle is 2°. The perfect individual design is calculated considering this value, and therefore will have the best possible optical performance for the wearer. The optimised design is calculated for a fixed position of wear. Under this assumption, the lens will be ideal for a wearer with an 8° pantoscopic angle (the average value for the present study) but will underperform when the lens is worn with a different pantoscopic tilt. For the individual design, we considered that the pantoscopic tilt was measured with some amount of error, perhaps obtaining a finding of 3.5°. This value will be entered into the calculation and the resulting lens will be ideal for 3.5° but again will underperform when the lens is worn by the wearer.

These calculations require the definition of a progressive surface for the conventional/basic design and a progressive target power map for the optimised, perfect individual and individual designs. For this purpose, we created a progressive power lens design in which the optical areas for the distance, intermediate and near zones were similar to general‐purpose PALs currently on the market.^15^, ^16^

**FIGURE 2:**
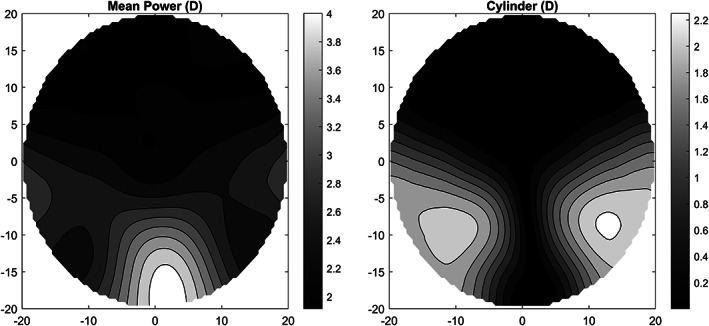
Target power map (left) and cylinder power (right) for a patient with +2.00 D sphere and +2.00 D addition.

In the case of the optimised, individual and perfect individual designs, the surface powers that define our design are used, along with the prescription, as targets for the user‐perceived power. This procedure delivers a lens in which oblique aberrations are minimal, and the actual power perceived by the user matches the design. As an example, Figure 2 shows a target power and cylinder maps for a patient with +2.00 D sphere and +2.00 D addition.

In the case of the conventional/basic design, we started with a design calculated with a plano distance prescription and the near addition required by the user. Then we superimposed the actual distance prescription of the patient, which should produce a design with the correct distance and near powers. By doing so, we emulate the construction of a conventional design, in which the lens is made from a fixed front surface that contains the near addition and a toroidal back surface.

Once the four lenses were calculated, we compared their performance by simulating the power perceived by the user assuming a lens‐eye system positioned in accordance with the true individual parameters.

Prescription and powers are represented by power vectors.^17^ Under this description, a prescription with sphere S, cylinder C and axis α was represented by vectors with components M, J_0_ and J_45_, calculated as (S + 0.5C), (−0.5C Cos 2α) and (−0.5C Sin 2α), respectively.

The terms p, $p_{F}$ and $p_{N}$ were adopted to refer to the user's prescription, the average power provided by the lens in a circle of 3 mm radius around the distance vision reference point (DRP) and the power provided by the lens in a circle of 3 mm radius around the near vision reference point (NRP), respectively. Each design will provide different values for $p_{F}$ and $p_{N}$, and for the perfect individual, $p≅p_{F}$ and $p+A\cdot i≅p_{N}$, where A is the prescribed near addition and $i=\left(1,0,0\right)$ is the power vector corresponding to a one‐dioptre sphere.

Now, we can characterise the performance of a given design by the error metrics:

(1) $$m_{F}=\left\|p_{F}-p\right\|,$$

and

(2) $$m_{N}=\left\|p_{N}-p-A\cdot i\right\|.$$

where the operator **|| ||** denotes the standard vector norm, that, when applied to Thibos vectors, provides dioptric distance.

For example, assume a patient with a +2.00 D spherical prescription and a true wrap angle of 15°. If we calculate the optimum design, then the calculation takes a fixed position of 5°. When the lens is simulated for the true position of wear, the user will not perceive +2.00 D but instead a different value, which we will assume to be +2.12 D with no cylinder, for simplicity. In this case the metric $m_{F}$ is $\left\|\left(2.12,0,0\right)-\left(2,0,0\right)\right\|=0.12\;D$.

## RESULTS

**FIGURE 3:**
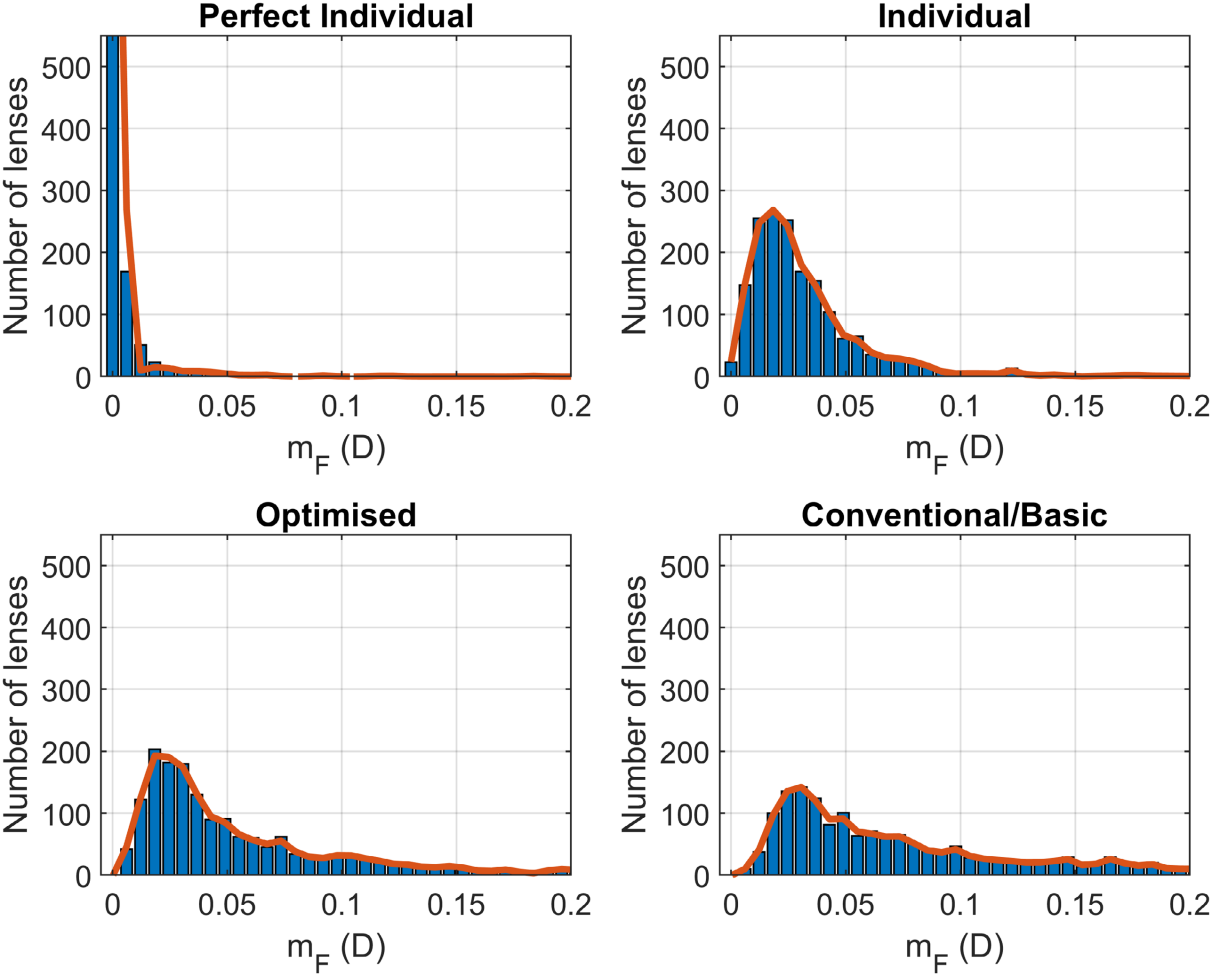
The distance metric ($m_{F}$) for each of the calculated designs.

After simulating and evaluating metrics (1) and (2) for each simulated user, we obtained a list of metric values for each design. Figure 3 shows $m_{F}$ for each of the four designs (blue bars). The red curve represents the smoothened histogram.

We see that the perfect individual gives a value close to $m_{F}=0$ for almost all of the lenses. The other three graphs display similar shapes, but the peak of the curve and the extension of the tail towards larger values of the error metric varies with the design.

**FIGURE 4:**
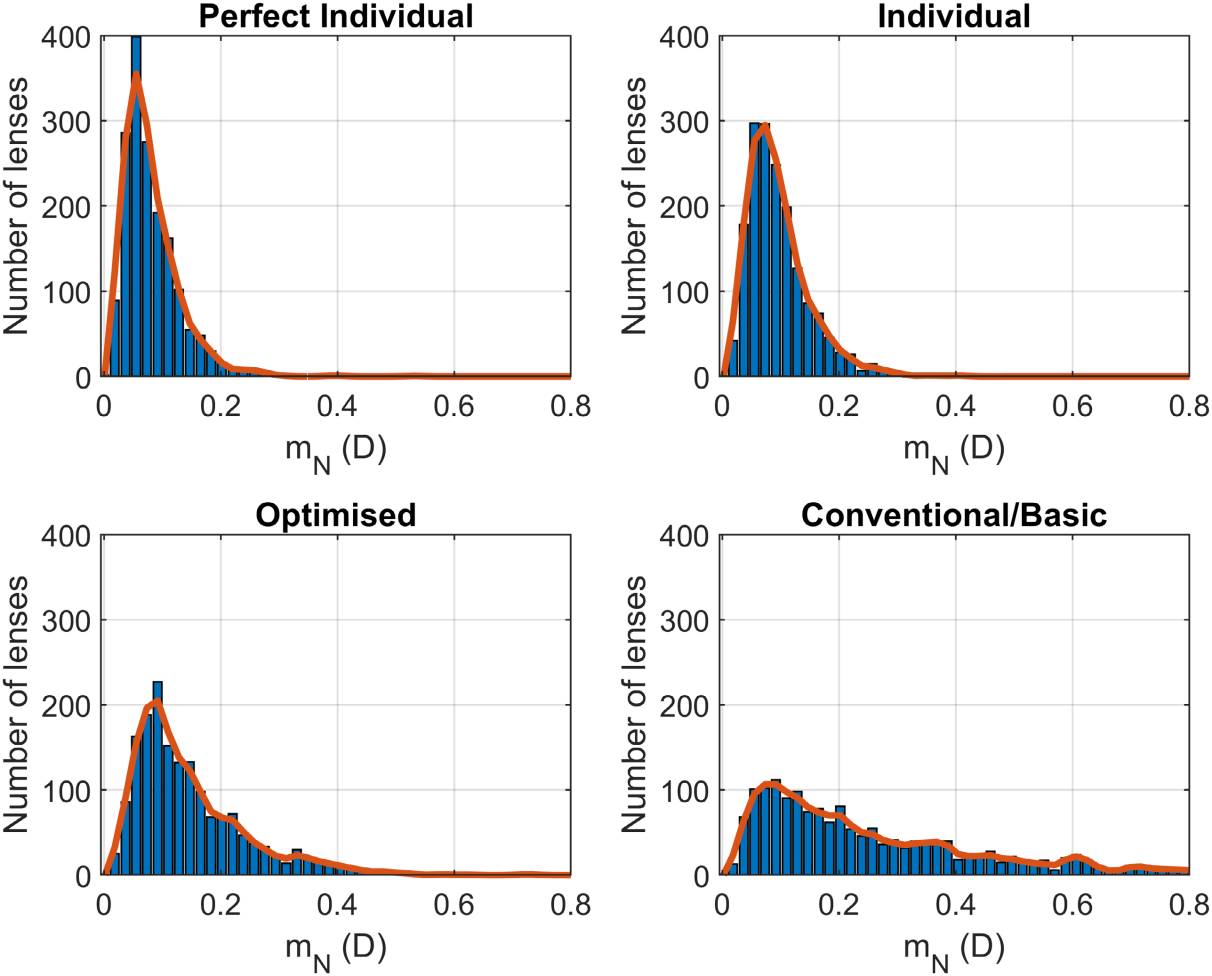
The near metric ($m_{N}$) for each of the calculated designs.

Similarly, Figure 4 shows $m_{N}$ for each of the four designs (blue bars). An interesting detail is that the perfect individual design presents a non‐negligible power error, which was not the case for the metric $m_{F}$. This might seem counterintuitive given that this design should be perfect by construction. There are two reasons for this error in $m_{N}$ in the perfect individual design. First, the lens is calculated to perform the best globally and to provide the exact user prescription at the DRP. The optimisation was designed to get $p_{N}$ right, but some error (always much smaller than the International Standards Organization / American National Standards Institute (ISO/ANSI) specifications^18^, ^19^) can be present because of optimisation balances. Second, and more important, $p_{N}$ is obtained as the power average in a finite‐size circle around the NRP. The addition typically peaks at the NRP, where astigmatism due to the Minkwitz theorem^3^ is at a minimum. As we consider points away from the NRP, the addition decreases, and Minkwitz astigmatism increases, thereby making the average power around the NRP slightly different from the power at the NRP.

PALs usually contain an extensive distance area, a narrow corridor and a limited near area. That means power is more stable around the DRP than around the NRP. This effect explains why, for the ‘perfect individual’ design, $m_{F}$ is virtually zero, whereas $m_{N}$ is not zero.

**FIGURE 5:**
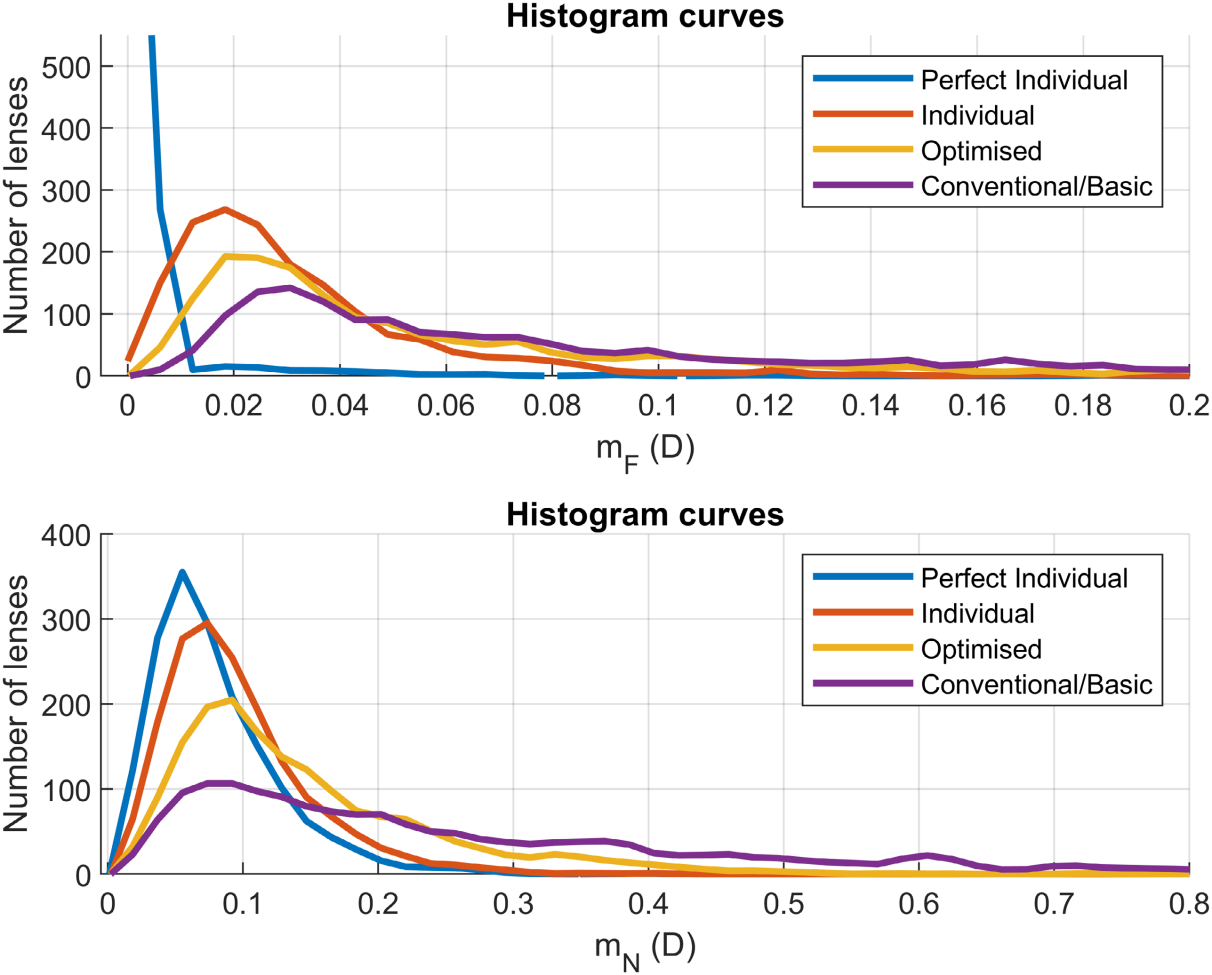
Super‐imposed histogram curves of the designs for the distance metric $m_{F}$ (upper figure) and near metric $m_{N}$ (lower figure).

Figure 5 shows the $m_{F}$ and the $m_{N}$ metrics for each of the four designs. When superimposing the curves, we see that the error grows in order from the perfect individual, to the individual, then the optimised and lastly the conventional design. We see this growth in two features of the curves: the position of the maximum and the tail towards larger metric values.

Qualitatively, we see a clear tendency towards optical degradation from the perfect individual to the conventional design. The mean and standard deviation of the corresponding distributions conveys the same conclusion, as shown in Table 1. However, the question arises as to whether this loss of optical performance is sufficient to be perceived by the user.

**TABLE 1:** Average and standard deviation (SD) values of the four designs for the distance ($m_{F}$) and near ($m_{N}$) metrics

Design	Average ($m_{F}$)	SD ($m_{F})$	Average ($m_{N}$)	SD ($m_{N})$
Perfect individual	0.00	0.01	0.08	0.05
Individual	0.03	0.03	0.10	0.05
Optimised	0.06	0.07	0.15	0.10
Conventional/basic	0.10	0.10	0.28	0.25

There is a relationship between the metrics $m_{F}$ and $m_{N}$ and the loss of visual acuity as shown by Raasch^20^ and Blendowske.^21^ A metric value of 0.25 D^22^ indicates a loss of decimal visual acuity of 0.1 in the corresponding area, which would be noticed by the wearer as a degradation in optical performance.

We computed the number of lenses in which either metric exceeded 0.25 D, as shown in Table 2. This provides an estimate of the percentage of users who would notice optical degradation at either the DRP or the NRP.

**TABLE 2:** Proportion of lenses with distance ($m_{F}$) and near ($m_{N}$) metrics above 0.25 D

Design	Lenses with $m_{F}$ > 0.25	Lenses with $m_{N}$ > 0.25
Perfect individual	0.00	0.01
Individual	0.00	0.02
Optimised	0.02	0.13
Conventional/basic	0.07	0.41

### Power segmentation

In this section, we present the correlation between the calculated metrics $m_{N}$ and $m_{F}$ and the lens prescription. We observed a correlation between $m_{N}$ and $m_{F}$ with the mean power at distance or near, but not with the cylinder alone or the addition alone. The strongest correlation ($r^{2}=0.25$ and *p*‐value ≤ 0.001) was between $m_{N}$ and the near mean power, and therefore we focused the analysis on these variables.

**FIGURE 6:**
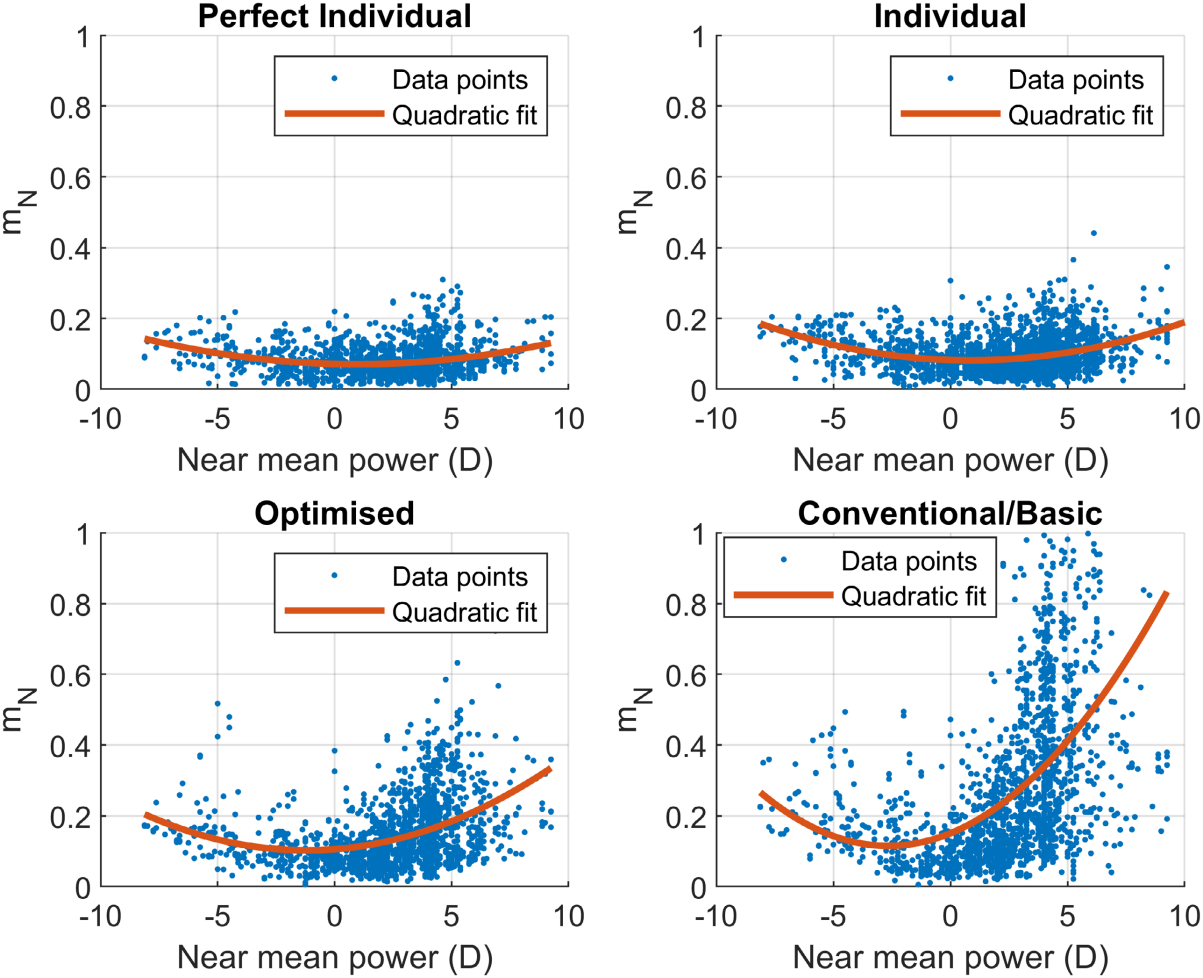
The near metric ($m_{N}$) plotted against the near mean power (blue dots) and a second‐order polynomial fit (red solid line) for each of the four lens designs.

The relationship between $m_{N}$ and the near mean power is shown in Figure 6. The distribution of $m_{N}$ is quite scattered, although we see a consistent growth of $m_{N}$ for high minus and plus powers. A second‐order polynomial fitting captures this growth towards higher powers. The estimated value and standard deviation of the second‐order coefficient are shown in Table 3. The *p*‐values in all cases are <0.001. Although the data points were highly scattered, the standard deviations are small compared with the estimated values, indicating that the second‐order fitting is correctly capturing the growth towards higher powers.

**TABLE 3:** Second‐order coefficient estimation and standard deviation (SD)

Design	Estimate	SD
Perfect individual	0.87	0.09
Individual	1.29	0.08
Optimised	2.13	0.17
Conventional/basic	5.07	0.41

*Note*: The units are $10^{-3}D^{-1}$.

**FIGURE 7:**
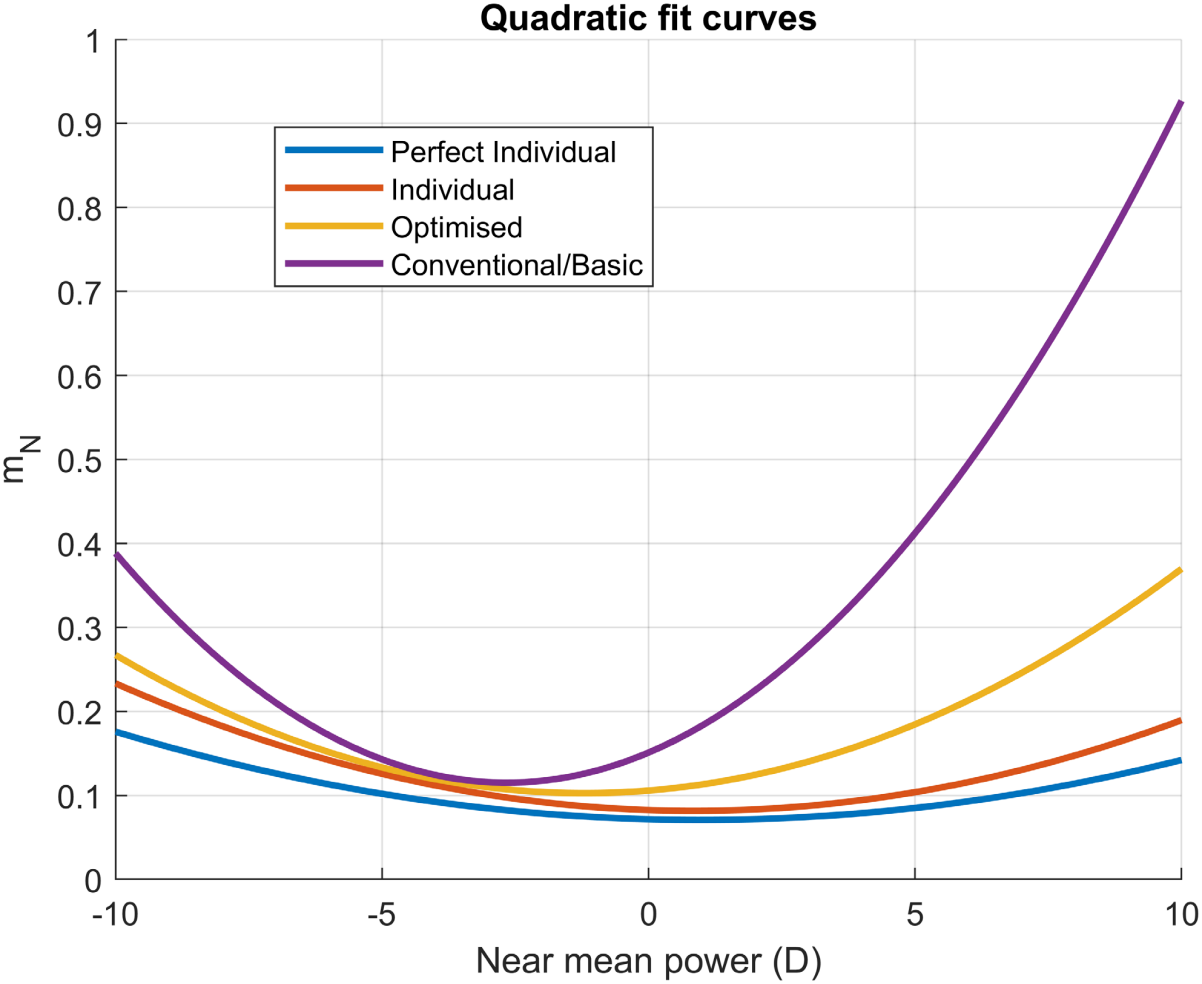
Polynomial best‐fit curves of the near metric ($m_{N}$) against the near mean power for each of the four designs.

Additionally, we see from Table 3 that the growth of the metric for higher values of the near power steepens as we move from the perfect individual to the conventional design. This is also seen when plotting the polynomial fits for the four designs, as shown in Figure 7. Further, the conventional design showed a very steep growth of $m_{N}$ for increasing plus powers, which was not seen with the other three designs.

Additionally, we segmented the mean near power $\left(P_{N}\right)$ between high minus $\left(P_{N}<-5\right)$, moderate minus $\left(-5<P_{N}<0\right)$, moderate plus $\left(0<P_{N}<5\right)$ and high plus $\left(P_{N}>5\right)$, and evaluated the average $m_{N}$ for these intervals. These results are shown in Table 4. The segmentation confirms the trends observed in Figure 7 both across power and lens designs.

**TABLE 4:** Average near metric ($m_{N}$) for each of the four designs segmented by power

Design	High minus	Moderate minus	Moderate plus	High plus
Perfect individual	0.11	0.08	0.07	0.09
Individual	0.15	0.09	0.09	0.12
Optimised	0.17	0.10	0.14	0.21
Conventional/basic	0.22	0.12	0.28	0.50

*Note*: High minus $\left(P_{N}<-5\right)$, moderate minus $\left(-5<P_{N}<0\right)$, moderate plus $\left(0<P_{N}<5\right)$ and high plus $\left(P_{N}>5\right).$

## DISCUSSION

### Individual versus perfect individual

The results show that the individual lens design has an increase in $m_{F}$ and $m_{N}$, of 0.03 and 0.02 D, respectively, compared with the perfect individual design. However, this increase in the average metric does not have a noticeable impact on visual acuity. Few users would notice a drop in visual acuity in the distance area, and only 2% of the users would notice a loss of visual acuity of 0.1 (decimal) at the NRP.

It is interesting to see that the distribution curves of the metrics for both the individual and the perfect individual designs have almost no noticeable tail towards high values. This indicates that the performance of both designs is very consistent across users, as there are no subsets exhibiting large values. We conclude that the individual design works almost as well as the perfect individual design, with only a small drop in visual performance.

### Optimised versus individual

In the case of the optimised design, we observed an increase of 0.06 and 0.07 D in $m_{F}$ and $m_{N}$ , respectively. More remarkably we found that 13% and 2% of the users would notice a respective loss of optical performance at near and far. Therefore, population‐wise, the performance of the optimised design is clearly worse than that of the individual design.

The longer tail of the distribution for the optimised design may be explained as follows. Whenever a user has at least one individual parameter that is significantly different from the averaged parameters, then the lens will noticeably underperform. This theoretical result is consistent with previous work. For example, Muždalo et al.^8^ concluded that the subjective quality and comfort of individual lenses were 12% greater than for optimised designs.

### Conventional/basic versus individual

This comparison shows an even greater reduction of optical performance for the conventional design, with almost 41% of users noticing some loss of visual acuity at near, and 7% at distance. A closer inspection of the conventional/basic design curve shown in Figure 5 indicates an important tail towards very high values of the metrics.

The explanation behind this behaviour is that the conventional design was calculated from a fixed surface in which the spherocylindrical prescription was superimposed on the addition. Therefore, there is no ray‐trace optimisation or minimisation of oblique aberrations, which will vary markedly with the prescription and base curve.

Accordingly, this design is expected to underperform severely in cases of moderate/high prescriptions and flat base curves, which account for most of the lenses. We can state that the individual and the optimised designs stand at a different quality level compared with the conventional design, as the visual performance is significantly increased when the prescription, base curve and position of wear (even if it is fixed) are considered during the optimisation.

Many previous works have studied the performance of conventional design PALs. Chamorro et al.^6^ noted that 63% of the users preferred individual over conventional lenses. Furthermore, Muschielok et al.^2^ concluded that individual PALs were rated more highly in terms of comfort and tolerability, while Han et al.^1^ showed that individual lenses were significantly preferred over conventional designs. Arroyo et al.^16^ extended Sheedy's scoring technique^15^ to compare the performance of individual and conventional designs, showing the superiority of the former lens type.

Although these results show that the conventional design has the worst performance, it should be mentioned that 59% of the users will not experience a noticeable loss of optical quality at near, which explains why conventional lenses have been used successfully for many years.

### Dependency on lens power

Figure 6 shows a clear significant correlation (*p* < 0.001) between $m_{N}$ and the lens near power, indicating a worse performance with high prescriptions. This behaviour is expected as it is known that oblique errors depend on the lens power.^23^

This dependency was observed by Han et al.,^2^ who showed a correlation between the preference for individual lenses over conventional lenses and the user's prescription.

Arroyo et al.^24^ noted that the performance of conventional designs had a strong dependency on the base curve since plus lenses are often made with base curves that are too flat compared with the optimum value (as derived from the Tscherning ellipse) due to aesthetic or practical reasons. This finding is consistent with the results presented in Table 4, indicating that the conventional design performs particularly badly for high‐plus lenses, with a $m_{N}$ value that is almost double the equivalent for high‐minus lenses.

### Interactions with other sources of errors

We calculated the metrics $m_{N}$ and $m_{F}$, assuming measurement errors in the individual parameters were the only ones affecting the lens. However, during the production of ophthalmic lenses, there are other errors that can affect the quality of the lens. Other error sources will be considered to assess the significance of the results shown.

Regarding possible measurement errors in the naso‐pupillary distance, several studies have shown that most devices exhibit good precision and repeatability when measuring this parameter. The typical standard deviations in this parameter are approximately 1.0 mm.^12^, ^14^ Note that we defined $m_{F}$ and $m_{N}$ as the average power inside a 3 mm radius around the DRP and NRP, respectively, so these metrics are being evaluated in an area much bigger than the typical error in the naso‐pupilary distance. Therefore, we can disregard the influence of this error.

Regarding manufacturing errors, we assumed a typical surfacing yield of 95%, meaning that 5% of the lenses lie outside the ISO^18^ tolerances at either the DRP or the NRP. For moderate powers, the ISO tolerance is ±0.12 D.^18^ We have simulated the effect of the manufacturing error by adding a normally distributed random power error such that the probability of being outside ISO tolerance is 5%.

Taking this error into consideration, we computed metrics $m_{F}^{\prime }$ and $m_{N}^{\prime }$, as shown in Table 5. The average values were increased only by a few hundredths of a dioptre with respect to those presented in Table 1, with $m_{F}^{\prime }$ for the perfect design being most affected. That is a logical outcome since the perfect individual started with an average $m_{F}$ of 0.00 D, so the addition of any other source of error will dominate $m_{F}^{\prime }$. In the other designs the increments were smaller, close to 0.03 D. This indicates that manufacturing error introduces a negligible increment to the metrics, particularly for $m_{N}^{\prime }$ . Table 6 shows the proportion of lenses for which either metric exceeded 0.25 D. Again, the increment due to the manufacturing error was negligible.

**TABLE 5:** Average and standard deviation (SD) values of the distance ($m_{F}^{\prime }$) and near ($m_{N}^{\prime }$) metrics considering manufacturing errors for the four lens designs

Design	Average ($m_{F}^{\prime }$)	SD ($m_{F}^{\prime }$)	Average ($m_{N}^{\prime }$)	SD ($m_{N}^{\prime })$
Perfect individual	0.05	0.04	0.10	0.05
Individual	0.06	0.04	0.11	0.05
Optimised	0.09	0.06	0.16	0.09
Conventional/basic	0.12	0.09	0.29	0.24

**TABLE 6:** Proportion of lenses with distance ($m_{F}^{\prime }$) and near ($m_{N}^{\prime }$) metrics considering manufacturing errors exceeding 0.25 D

Design	Lenses with $m_{F}^{\prime }$ > 0.25	Lenses with $m_{N}^{\prime }$ > 0.25
Perfect individual	0.00	0.01
Individual	0.00	0.02
Optimised	0.03	0.15
Conventional/basic	0.08	0.43

## CONCLUSIONS

In recent years, we have seen an increase in the complexity and individualisation of PALs. While it is known that individual designs perform better than conventional lenses, it is unclear how much improvement occurs with different levels of complexity.

Moreover, it is known that measurements of individual patient parameters are less repeatable and more prone to errors than quantification of the naso‐pupillary distance. The impact of these measurement errors is unknown and may lead ECPs to disregard the use of individual designs.

This work compared four PAL designs: conventional, optimised, individual (with poorly measured parameters) and perfect individual. We showed that the optimised and individual designs are superior to the conventional form and exhibit superior optical performance. That is because optimised designs consider both the position of wear and minimisation of oblique aberrations, whereas conventional designs do not.

Furthermore, optimised designs perform worse than both the individual and the perfect individual lenses. This is because the optimised design uses a fixed position of wear, which works well for the majority of the users but underperforms for a non‐negligible minority of the population.

Finally, we showed that the individual design with poorly measured parameters performs similarly to the perfect individual design. This result indicates that the individual design has the best optical performance, better than the conventional and optimised designs, even when individual parameters are measured poorly.

## AUTHOR CONTRIBUTIONS

**Eduardo Pascual:** Conceptualization (lead); data curation (lead); formal analysis (lead); investigation (lead); methodology (lead); project administration (lead); resources (lead); software (lead); visualization (lead); writing – original draft (lead). **José A. Gómez‐Pedrero:** Conceptualization (supporting); data curation (supporting); formal analysis (supporting); funding acquisition (equal); investigation (supporting); methodology (supporting); project administration (supporting); resources (supporting); supervision (equal); validation (equal); visualization (supporting); writing – review and editing (equal). **José Alonso:** Conceptualization (supporting); data curation (supporting); formal analysis (supporting); funding acquisition (equal); investigation (supporting); methodology (supporting); project administration (supporting); resources (supporting); supervision (equal); validation (equal); visualization (supporting); writing – review and editing (equal).

## CONFLICT OF INTEREST

The authors declare no conflicts of interest.
